# Who Denigrates Today’s Youth?: The Role of Age, Implicit Theories, and Sharing the Same Negative Trait

**DOI:** 10.3389/fpsyg.2022.723515

**Published:** 2022-05-30

**Authors:** John Protzko, Jonathan W. Schooler

**Affiliations:** ^1^Central Connecticut State University, New Britain, CT, United States; ^2^Department of Psychological and Brain Sciences, University of California, Santa Barbara, Santa Barbara, CA, United States

**Keywords:** social cognition, prejudice, stereotyping, implicit theories of change, implicit theories of personality, children, adult personality development, implicit change

## Abstract

Adults perceive the youth of the present as being worse than from when they were young. This phenomenon has been shown to be a product of a memory bias, adults are unable to accurately recall what children were like in the past so they impose their current selves onto their memories. In two studies using American adults (*N* = 2,764), we seek to connect this finding to age, implicit theories of change, and extend the beliefs in the decline of the youth to new domains. Here we show as people age, they hold harsher beliefs about present children. Those who believe a trait does not change throughout the lifespan exhibit more forgiving attitudes toward the youth of today, believing they may not be in such decline *on that trait*. Finally, people who are low in a negative trait believe strongly that children are becoming more deficient in that particular trait (e.g., those who are not narcissistic believe the youth are becoming more narcissistic).

## Introduction

“Kids, they are disobedient, disrespectful oafs; noisy, crazy, sloppy, lazy loafers…why can’t they be like we were, perfect in every way? What’s the matter with kids today?” (Bye Bye Sidney, 1963).

People widely believe that children of the present are in decline along a number of dimensions. Previous work has shown a driving force of this belief is a memory bias for the past; one where we impose our current selves onto our memories for what past children were like (Protzko and Schooler, 2019). But people themselves are not stable. We grow, decline, and change in some ways—while in other ways we can be surprisingly stable. Here, we investigate the relationship between believing children are in decline and beliefs about how those supposedly declining traits change. If people believe a trait changes over the lifespan (vs. believing it is stable), they may hold different beliefs about intergenerational change. We further investigate how both beliefs about children and traits vary as a function of age, to see how such prejudice may change over the lifespan.

### Kids These Days!

People who are more intelligent think children are becoming less intelligent, people who respect authority believe children are becoming less respectful of authority, people who are well-read think children enjoy reading less (Protzko and Schooler, 2019). This phenomenon, called the kids these days effect (KTD effect), is the tendency to believe children of the present are failing on those traits one happens to be high on due to a biased memory mechanism. For each trait that was previously investigated, however, being high in the trait is a good thing (being intelligent, respectful, and well-read).

One of the mechanisms through which the KTD effect operates is imposing one’s current standing on a trait backward in time to children of the past. Meaning, one who is well-read believes children “in their day” were well-read and therefore present children cannot compare to this elevated past; those who are not well-read do not hold such views (Protzko and Schooler, 2019). This process should therefore also occur when the trait in question is negative; someone who is currently *low* in a negative trait, such as entitlement, would believe children of the past were not entitled and “see” children today as *more* entitled than past children. In this study, we investigate whether the KTD effect also applies to negative traits.

### Implicit Theories of Stability and Change

We are not the same as we were as children, nor will we be the same as elders. Sometimes, this is for the better, other times, for the worse. Our intuitions about how we have and will change is filled with errors [e.g., Quoidbach et al. (2013)], yet we have these implicit theories about stability and change nonetheless (Ross, 1989). Implicit theories of trait changes over time entail more than the simple fixed/growth dichotomy [e.g., Dweck (2008)], as traits can evolve in a variety of ways, including a parabolic increase into adulthood and then a decrease into old age, or increasing from childhood but then plateauing, or a U-shaped decrease from childhood into adulthood followed by an increase in old age. Do people hold the same implicit beliefs about how a trait changes over time, and are these beliefs of change related to what extent they denigrate the youth?

If people believe that a trait is fixed (i.e., that they are the same as they and *everyone* is the same as they have always been on a trait) then they may be more forgiving toward present children. A general belief that a trait is fixed within a lifetime may contribute to a perception of relatively little inter-generational change, and therefore that children today are no different on that trait than previous generations. Believing there is room for change and growth in a trait, however, may open room for differences across generations. A trait that is seen as unstable may be interpreted as something that simply changes in a “natural” fashion.

Here we investigate, in two studies, whether the KTD effects extend to new traits, including negative traits, and how these beliefs in the failings of the youth are associated with implicit theories of change.

## Study 1

In study 1 we take two main approaches. The first is to investigate the KTD effect in two new traits, self-reliance and entitlement. The trait of entitlement is of particular interest because it provides the first examination of the KTD effect in regards to a negative trait. The second approach is to examine the relationship between the belief that children are in decline and people’s intuitive theories of change about those traits. As an auxiliary investigation, we also explored how age was related to both implicit theories of change and the denigration of the youth. This study was pre-registered prior to data collection (see Supplementary Appendix for links).

### Materials and Methods

#### Participants

Participants were 1,264 American adults (Age range 18–90 years old) drawn from a proprietary internet panel. The panel was instructed to draw the sample in a stratified way with unequal probabilities of selection, so that the people who complete each survey will resemble the nation’s adult population (according to the most recently available Current Population Survey, conducted by the U.S. Census Bureau) in terms of gender, age, education, ethnicity (Hispanic vs. not), race (allowing each respondent to select more than one race), region, and income. These demographics were controlled by the panel provider and not asked by us, except for sex (our sample was 53% female). We aimed for 1,500 participants but due to 16% of our sample failing a comprehension check (see below) we ended up with fewer. Participants were first asked their age (M = 51.32, SD = 15.67 range 18–93). Then, were asked about declines in children or implicit theories of change scales in random order.

#### Belief in the Decline of Children

##### Measures

All participants first read: “We would like to know your thoughts about children. Compared to when you were a child: Do you think children today are (more trait/less trait/equally trait) as children were when you were a child?” For the traits in question, we used the following: intelligent, enjoy reading, respectful of their elders, able to stay focused, delaying gratification, able to save money, work ethic, self-sufficient, morally good, and entitled. Response options were “more/better trait; equally trait; worse/less trait.” All questions were presented in random order (see link in Supplementary Appendix for full question wordings).

Afterward, on a separate page, for each question about ‘kids these days’ participants answered “more than” or “less than” to, they read the appropriate versions of the following: “How much more/less (trait) are children now compared to when you were a child?” Response options were unnumbered a lot more/less = −3/somewhat more/less = −2/a little more/less = −1. All questions were in random order.

###### Entitled and Self-Sufficient

We administered the entitlement and self-sufficiency subscales from the Narcissism Personality Inventory (Kubarych et al., 2004). This was to test the prediction that more self-sufficient people think “kids these days” are becoming less self-sufficient and that more entitled people think “kids these days” are becoming less entitled. All items were administered in random order with unnumbered response options in random order. As all items come from the overall Narcissistic Personality inventory, they were on the same page.

All participants first read:

“This inventory consists of a number of pairs of statements with which you may or may not identify. Consider this example: I like having authority over people/I don’t mind following orders. Which of these two statements is closer to your own feelings about yourself? If you identify more with “liking to have authority over people” than with “not minding following orders,” then you would choose that option. You may identify with both options. In this case you should choose the statement which seems closer to yourself. Or, if you do not identify with either statement, select the one which is least objectionable or remote. In other words, read each pair of statements and then choose the one that is closer to your own feelings. Indicate your answer by selecting the item. Please do not skip any items.”

See Table 1 for items.

**TABLE 1:** Items used for measuring entitlement and self-sufficiency.

**Entitled sub-scale**	
I like to take responsibility for making decisions.	If I feel competent I am willing to take responsibility for making decisions.
I always know what I am doing.	Sometimes I am not sure of what I am doing.
I rarely depend on anyone else to get things done.	I sometimes depend on people to get things done.
I can live my life in any way I want to.	People can’t always live their lives in terms of what they want.
I am going to be a great person.	I hope I am going to be successful.
I am more capable than other people	There is a lot that I can learn from other people.
**Self-sufficiency**	
I have a natural talent for influencing people.	I am not good at influencing people.
If I ruled the world it would be a better place.	The thought of ruling the world frightens the hell out of me.
I see myself as a good leader.	I am not sure if I would make a good leader.
I like to have authority over other people.	I don’t mind following orders.
I find it easy to manipulate people.	I don’t like it when I find myself manipulating people.
I will never be satisfied until I get all that I deserve.	I take my satisfactions as they come.
I have a strong will to power.	Power for its own sake doesn’t interest me.
People always seem to recognize my authority.	Being an authority doesn’t mean that much to me.
I would prefer to be a leader.	It makes little difference to me whether I am a leader or not.
I am a born leader.	Leadership is a quality that takes a long time to develop.

*Participants score 1 point for each option in the right-hand column. Items presented in random order with response options in random order.*

#### Implicit Theories of Change

**FIGURE 1:**
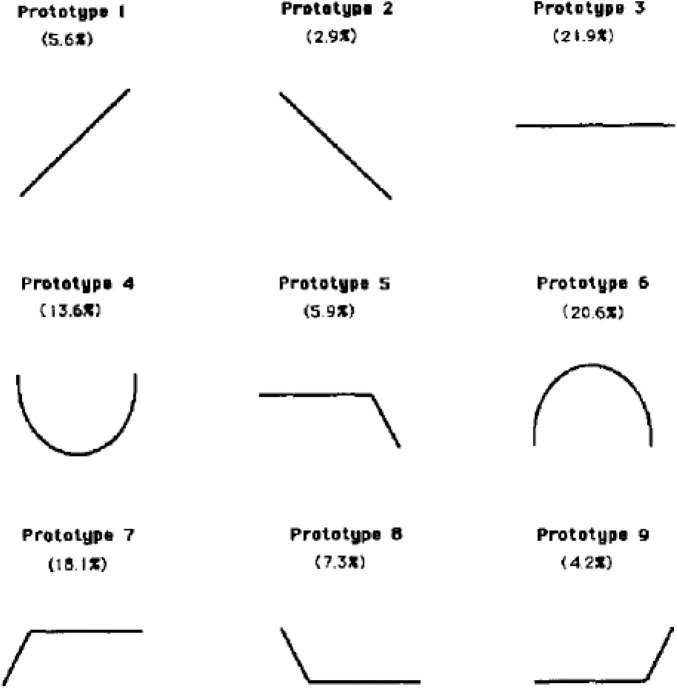
Images representing different implicit theories of developmental change. Image taken from Figure 1 of Ross (1989). Participants saw each line with a description. Patterns referred to here as (in numerical order): increasing, decreasing, flat, U, elderly decline, parabolic, plateau, floor, elderly spike.

For the implicit theories of change questions, participants first saw a page with nine images presented horizontally all with a width of 125 pixels (see Figure 1).

To ensure participants understood the different natures of the change processes, we explained to them:

“On the next pages we will ask you about what you think happens to a number of different traits as we age and grow older. Below you will see nine different graphs. Each graph represents one pattern of what happens to a trait as we age. As an example, let’s use how religious someone is. The first graph would mean you think people get more and more religious as they age. The second graph would mean you think people get less and less religious as they age. The third graph would mean you think people do not change in how religious they are as they age. The fourth graph would mean you think people start off religious as young children, get less religious through adulthood, but then get more religious as they grow into old age. The fifth graph would mean you think people don’t change in how religious they are for most of their life, but then become less religious in old age. The sixth graph would mean you think people are not religious as children, they become more religious as they grow into adulthood, and then become less religious as they enter old age. The seventh graph would mean you think children are not religious, they become more religious as they age, then it does not change in adulthood through old age. The eighth graph would mean you think people start off religious as children, but eventually become less religious and never become more religious again. The ninth graph would mean you think people are not religious for most of their lives, but then become religious as they enter older ages. What do you think happens to how religious people are throughout their lives? Please select the option that most closely matches what you think happens.”

Then, on the next page, participants saw the same nine images, this time presented vertically and in random order, and read: “Thank you, on the next pages you will be asked about your beliefs in change over the life on a number of different topics. To make sure you fully understand how to answer, please select below the option that shows no change whatsoever across the life.” Any participant who failed this simple comprehension check was excluded from the analyses (consistent with our pre-registration).

Then, in random order, one page at a time, participants were asked about their belief in change on a number of traits. The order of pictures for each trait was presented vertically in random order. Participants were asked: “Starting from childhood and extending into old age, what do you believe happens to (trait) as we age? What picture best represents how we increase, decrease, and stay stable over the lifespan?” The traits we asked about were: intelligence, respect for authority, enjoying reading, ability to focus on one thing, ability to delay gratification, morality, ability to save money, work ethic, being self-sufficient, and entitlement.

#### Modeling Strategy

Our pre-registered analysis strategy involved first focusing just on the association of implicit theories of change with beliefs in the decline of the youth, before moving onto an analysis including age (page with links in the Supplementary Appendix). The original plan was then to add in age into the analysis as a covariate. These analyses are simultaneously largely consistent with the results we report below, but also not an accurate representation of the phenomenon under study. This is because the implicit theories of traits change people hold are not the same for older and younger participants. Older people do not have the same implicit theory of change for the same trait as younger people. Therefore, a simple linear regression would not capture the relationship of age on implicit theories, and a moderation would inappropriately omit the relationship between shifting implicit theories over age. We therefore believe the optimal model is similar to that of an indirect effects design, with age predicting both belief in the degradation of children (the *c*’ path) and implicit theories of change (the *a* path). Therefore, in one model, we can look at how implicit theories of change develop over the lifespan across traits and how they simultaneously relate to belief that children are in decline. Our focus is then on what could be considered the *b* paths from an indirect effects design—the relationship of implicit theories on belief in the decline of children, after conditioning both variables on age.

In this modeling strategy, we first show what would be considered the direct effect of age on beliefs of the decline of children (the *c* path). Then, we describe how implicit theories of change develop over the lifespan for the different traits. Finally, we report the full structural equation modeling results that place into context how implicit theories of change relate to the belief in the decline of children.

### Results

#### Beliefs of the Decline of the Youth With Age

The older someone is, the more they believe youth of the day are becoming deficient in all of the traits (see Supplementary Appendix for link to all results). Compared to younger participants, older people believe the youth of today are becoming less intelligent (*b* = −0.005, *p* = 0.096, 95%CI = −0.01–0.01), less respectful of authority (*b* = 0.015, *p* < 0.001, 95%CI = 0.02–0.01), enjoy reading less (*b* = 0.015, *p* < 0.001, 95%CI = 0.02–0.01), are less able to focus on one thing (*b* = 0.007, *p* = 0.013, 95%CI = 0.013–0.002), are less able to delay gratification (*b* = 0.018, *p* < 0.001, 95%CI = 0.023–0.013), are less moral (*b* = 0.009, *p* = 0.002, 95%CI = 0.014–0.003), are less able to save money (*b* = 0.017, *p* < 0.001, 95%CI = 0.022–0.012), have less work ethic (*b* = 0.019, *p* < 0.001, 95%CI = 0.024–0.013), are less self-sufficient (*b* = 0.011, *p* = 0.001, 95%CI = 0.017–0.004), are *more* entitled (*b* = 0.014, *p* = 0.001, 95%CI = 0.022–0.006). Thus, older people think the youth today are declining in every way we investigated.

#### “Kids These Days” Self-Sufficiency and Entitlement

We next investigated whether the kids these days effect could be shown with the positive trait of self-sufficient and the negative trait of entitlement. In the pre-registered model, there was not a statistically significant relationship between scores on the self-sufficiency subtest and believing children today are less reliant than children of the past were (β = −0.035, *p* = 0.178, 95%CI = −0.086–0.016); this model was particularly poorly fitting as well (CFI = 0.699, RMSEA = 0.059). We dropped the self-sufficiency term and just tested entitlement in isolation, which returned an exceptionally well-fitting model (CFI = 1, RMSEA = 0). With this improved model, the more entitled someone is, the more they believe that the youth today are becoming less entitled (β = −0.056, *p* = 0.029, 95%CI = −0.106 to -0.006). The corollary is the less entitled someone is, the more they believe children are entitled. This pattern of results is therefore consistent with what has been observed for “positive” traits. The higher you are on that trait, the less you think children today have it compared to children when you were a child.

#### Relationship Between Implicit Change Theories and the Kids These Days Beliefs

How do people’s beliefs in implicit change relate to their beliefs that children today are in decline? First, participants did not hold the same theories of implicit change for all traits (see Supplementary Appendix). Some people tended to think certain traits are parabolic over the lifespan (like self-sufficiency or the ability to focus on just one thing) while other traits develop through childhood but then plateau in adulthood (like work ethic). For yet other traits, people tended to think they are unchanging throughout the lifespan (like enjoyment of reading).

Complicating this, these implicit theories themselves varied as a function of the age of the respondent. Older participants had different intuitive beliefs about traits than younger participants. Therefore, to analyze the relationship between implicit theories and beliefs that children today are in decline across traits, while taking into account the fact that older individuals tend to view present children more negatively and their beliefs of implicit theories of change develop, we construct an indirect effects design. Here age is associated with both the pattern of implicit change (what would be the *a* path in an indirect effects design) and the belief that children are in decline (what would be a *c*’ path). Instead of looking at an indirect effect, however, we are looking at the residual relationship of implicit theories of change on the belief in the decline of children (what would be the *b* path in an indirect effects design). We arbitrarily chose “no change” as the reference variable, and provided dummy codes for all of the other theories of change. Therefore, the intercept corresponds to the belief that children today are in decline on that trait, conditioning on age, holding the implicit theory that the trait does not change. The coefficients then correspond to the relationship of having different implicit change beliefs, which themselves are different for different ages, on the belief in the decline of the youth.

We present the results by lay belief of change, indicating its relationship with each of the traits. This helps illustrate issues such as which beliefs were associated with the KTD effect across traits. Put another way, the beliefs are conceptually distinct with respect to KTD, the traits we assume going into it should all behave similarly. In most cases, believing that a trait is fixed throughout the lifespan is associated with the least amount of belief that children are in decline on that trait. For full analyses and results, and all other statistical output, see link in Supplementary Appendix.

##### Trait Increasing Throughout the Lifespan

Believing the ability to delay gratification increases throughout the lifespan is associated with believing that the youth today cannot delay gratification (*b* = 0.492, *p* = 0.001, 95%CI = 0.789–0.201), compared to believing the ability to delay gratification is stable. Those who believe morality increases throughout the life believe the youth today are becoming less moral (*b* = 0.366, *p* = 0.007, 95%CI = 0.63–0.1). Likewise, believing work ethic increases throughout the lifespan was related to having even stronger views that children today’s work ethic is in decline (*b* = 0.306, *p* = 0.032, 95%CI = 0.589–0.024). Finally, believing self-sufficiency increases throughout the lifespan was associated with stronger beliefs that children today are not as self-reliant as children used to be (*b* = 0.508, *p* = 0.04, 95%CI = 1.003–0.02).

##### Trait Decreasing Throughout the Lifespan

Believing the ability to delay gratification decreases throughout the lifespan is associated with believing that the youth today cannot delay gratification (*b* = 0.536, *p* = 0.01, 95%CI = 0.935–0.114), compared to believing the ability to delay gratification is stable. Likewise, believing morality decreases throughout the life is associated with stronger beliefs that the youth today are becoming less moral than they used to be (*b* = 0.577, *p* = 0.005, 95%CI = 0.978–0.175).

##### Trait Showing a U-Shape Throughout the Lifespan

Believing morality shows a U-shaped pattern over the life is related to stronger beliefs that the youth are becoming less moral (*b* = 0.395, *p* = 0.01, 95%CI = 0.692–0.091).

##### Trait Stable Until a Decline in Old Age

Believing morality is stable throughout life but declines in the elderly is associated with stronger beliefs that the youth are becoming less moral (*b* = 0.532, *p* = 0.008, 95%CI = 0.928–0.136).

#### Trait Showing a Parabolic Shape Throughout the Lifespan

Those who believe the ability to focus is parabolic hold even stronger views that children today cannot focus as well as children of the past (*b* = 0.384, *p* = 0.015, 95%CI = 0.693–0.074). Similarly, believing morality is parabolic is associated with holding stronger beliefs that the youth are becoming less moral (*b* = 0.371, *p* = 0.044, 95%CI = 0.721–0.009).

##### Trait Increasing Into Adulthood, Then Plateaus

Believing the ability to delay gratification grows until a plateau in adulthood is associated with stronger beliefs that the youth today cannot delay gratification (*b* = 0.432, *p* = 0.005, 95%CI = 0.739–0.133), compared to believing the ability to delay gratification is stable. Similarly, holding the belief that the ability to save money develops through childhood but then plateaus in adulthood throughout life is related to holding stronger beliefs that children today cannot save money like they used to be able to (*b* = 0.341, *p* = 0.043, 95%CI = 0.671–0.006).

##### Trait Decreasing Into Adulthood, Then Stable

Believing morality declines to an adulthood floor, then is stable is associated with believing the youth today are becoming less moral than they used to be (*b* = 0.638, *p* = 0.001, 95%CI = 0.998–0.264).

##### Trait Stable Until Old Age, Then Increases

People who believe that intelligence spikes upward in the elderly were more likely to believe that children today are becoming less intelligent (*b* = 0.455, *p* = 0.028, 95%CI = 0.848–0.036). Believing the ability to delay gratification spikes upward in adulthood is also associated with stronger beliefs that the youth today cannot delay gratification (*b* = 0.342, *p* = 0.032, 95%CI = 0.656–0.035). This pattern is also seen for morality (*b* = 0.401 *p* = 0.013, 95%CI = 0.702–0.078) and the ability to save money; those who believe the ability to save money increases in late adulthood are more likely to believe the youth today cannot save money like they used to (*b* = 0.513, *p* = 0.002, 95%CI = 0.84–0.188).

### Discussion

Study 1 showed five important things. First, the KTD effect was replicated in a new domain, entitlement, which is important both because it further establishes the breadth of the phenomenon and it generalizes it to a trait that many would consider negative. A major focus of Study 2 is to expand the investigation of the KTD effect into more negative traits, to see if we can replicate this finding that those *low* in a negative trait are *more* likely to see youth of the day getting worse in it. Second, Study 1 demonstrated that overall people think kids these days are in decline across a host of different traits not investigated before. Third it showed that this perception of decline was particularly pronounced with the elderly. Fourth it showed that theories of decline interacted with lay theories of change. Finally, it demonstrated that the nature of people’s theories varied both across individual and across traits. One focus of study 2 is to manipulate implicit theories of change to test the causal role of such beliefs on denigrating children.

## Study 2

Study 2 expands the investigation of why people believe children are in decline to additional traits that are considered negative. Previous work has shown people believe children of the present (regardless of which present it is) possess less admirable qualities than children of their past (Protzko and Schooler, 2019). Could it be the case that children are simply less of everything, including negative traits (e.g., children of the present are seen as less manipulative), or is it that people believe children of “the present” have less of good qualities but also more negative qualities?

### Procedures and Methods

Participants were 1,500 participants, drawn in a stratified way using the same sampling requirements in study 1. This sample size was chosen to maximize sample size under a fixed availability of funds to spend on this project.

Participants first filled out their age. They were then randomly assigned to either fill out the trait measures first or the kids these days scales first.

#### Trait Measures

All trait measures were administered in random order with each scale on a separate page. We chose a trait (self-control) that can be portrayed either positively (restraint) or negatively (impulsive). We also chose negative traits based on the literature of the Dark Triad [psychopathy, Machiavellianism, narcissism; Paulhus and Williams (2002)] and replicated our results from study 1 on entitlement. For each trait measure, we attempted to identify the most psychometrically sound scale possible.

##### Impulsivity and Restraint (Self-Control)

Self-control is the ability to shape one’s behavior through thoughtful behavior control [e.g., Carver (2005)]. Objective measures of self-control have shown poor measurement properties when administered online, most self-report measures also showing less than ideal measurement properties (Enkavi et al., 2019). One measure, however, has shown adequate measurement properties when administered online, the Brief-Self-control Scale [Maloney et al. (2012); see also Enkavi et al. (2019)]. This scale is composed of two subscales, one about how impulsive one is, and the other about how restrained one is. For our investigation we used both subscales (see Supplementary Appendix for full question wording and scoring). Reliability in our sample was good for the two-factor correlated factor model [P_*CF*_ = 0.85; Cho (2016, 2022)].

##### Psychopathy

Psychopathy is a trait conceptualized as impulsive behavior undertaken to gratify one’s desires with a complete lack of care for the impact on others [e.g., Hare et al. (1991)]. People who are high in psychopathy do not care about the consequences of their actions on other people. We chose the psychopathy scale from the dirty dozen scale (Webster and Jonason, 2013) as our measure of psychopathy (see Supplementary Appendix for full Wording and scoring). Reliability in our sample was good (ω_*p*_ = 0.88).

##### Machiavellianism

Machiavellianism is the trait of manipulating others toward your own ends [e.g., Paulhus and Williams (2002)]. For this scale, we likewise chose the Machiavellianism subscale from the dirty dozen measure (Webster and Jonason, 2013; see Supplementary Appendix for full question wording and scoring). Reliability in our sample was good (ω_*p*_ = 0.9).

##### Narcissism

While a narcissism measure also exists as part of the dirty dozen, investigations into the psychometric properties of that scale have cast doubt on the validity of the narcissism items [see Kajonius et al. (2016)]. To measure narcissism, we therefore administered the short Narcissistic Personality Inventory (Ames et al., 2006; see Supplementary Appendix for full question wording and scoring). The reliability of this scale in our study was good ($\hat{\omega }$ = 0.72, Padilla and Divers, 2016).

##### Entitlement

Entitlement was measured the same way as it was in study 1. The reliability of this scale in our study was adequate ($\hat{\omega }$ = 0.68, Padilla and Divers, 2016).

### Kids These Days Questions

Kids these days questions were measured using the same format as in study 1. All participants were first told “We would like to know your thoughts about children.” For self-control, we asked participants whether they think children today were better, worse, or equally able to control themselves as children could when they were a child. For psychopathy, we asked participants whether they think children today are more, less, or equally concerned about the morality of their actions as children were when they were a child. For Machiavellianism, we asked participants whether they think children today are less, more, or equally manipulative as children were when they were a child. For narcissism, we asked participants whether they think children today are less, more, or equally narcissistic as children were when they were a child. For entitlement, we asked whether participants think children today are more, less, or equally as entitled as children were when they were a child. All questions were presented on the same page in random order. Participants who chose “more than” or “less than” were asked on the next page to what extent they thought children were more or less on that trait. All scales were coded on -3 to 3 scales with 0 as “equal to” and higher scores indicating a stronger belief that children are declining.

We further attempted to manipulate implicit theories of change, to test the causal effects of such beliefs. This manipulation, however, proved unsuccessful, but additional details are available at the link in the (Supplementary Appendix). This study was pre-registered prior to data collection.

### Results

#### Self-Control

On average, people believed children today are worse at exerting self-control than children were when they were a child (*b*_0_ = 2.256, *p* < 0.001, 2.622–1.889). When it came to the two measures of participants’ self-control, our pre-registered model used both impulsivity and restraint as predictors simultaneously. Consistent with our theoretical model, the less impulsivity problems someone has, the more they believe that children today are worse at self-control (β = −0.296, *p* < 0.001). There was no relationship between how much restraint who believes they have and the belief that children today are in decline in self-control (β = −0.052, *p* = 0.135). Thus, the more impulsive (negative trait) someone is, the less they believe children today cannot control themselves. As an exploratory analysis we also found the older someone is the more they believe that children cannot control themselves (β = 0.328, *p* < 0.001).

#### Psychopathy

Similar to impulsivity, people believed that children today are more psychotic than children were when they were a child (*b*_0_ = 2.022, *p* < 0.001, 95%CI = 2.217–1.828). Furthermore, consistent with our predictions, the less psychopathic tendencies someone has, the stronger they believe children today are becoming more psychotic (β = −0.345, *p* < 0.001). As an exploratory analysis we also found the older someone is the more they believe that children are getting more psychopathic (β = 0.326, *p* < 0.001).

#### Machiavellianism/Manipulativeness

On average, people believed children today are becoming more manipulative than children were when they were a child (*b*_0_ = 1.392, *p* < 0.001, 95%CI = 1.573–1.211). Again consistent with our theoretical model, the less manipulative someone is, the more they believe children today are becoming more manipulative (β = −0.155, *p* < 0.001). As an exploratory analysis we also found the older someone is the more they believe that children are becoming more manipulative (β = 0.214, *p* < 0.001).

#### Narcissism

On average, people believed that children today are a little more narcissistic than children were when they were a child (*b*_0_ = 1.288, *p* < 0.001, 1.423–1.152). Consistent with our theoretical model, the less narcissistic someone is, the more they believe children are becoming more narcissistic (β = −0.131, *p* < 0.001). As an exploratory analysis we also found the older someone is the more they believe that children are becoming more narcissistic (β = 0.264, *p* < 0.001).

#### Entitled

Replicating study 1, people thought children today are a little more entitled than children were when they were a child (*b*_0_ = 1.45, *p* < 0.001, 95%CI = 1.59–1.31). Again consistent with our theoretical model, the less entitled someone is, the more they believe children today are becoming more entitled (β = −0.11, *p* < 0.001). As an exploratory analysis we also found the older someone is the more they believe that children are becoming more entitled (β = 0.33, *p* < 0.001).

**FIGURE 2:**
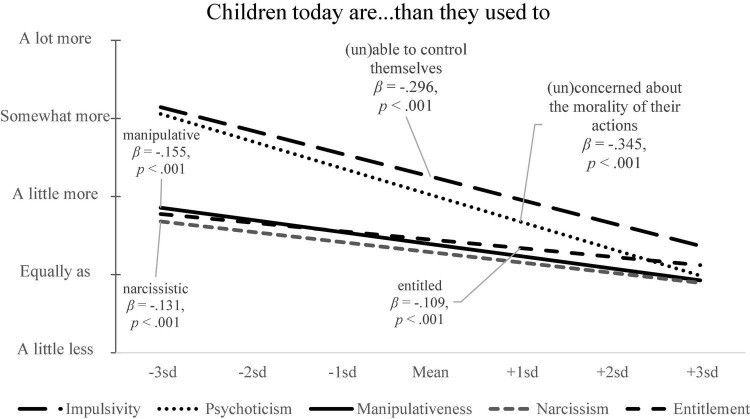
One’s standing on a trait and the belief that children today are particularly deficient in that one trait. The lower one is on a negative trait, the more likely they believe children today to be afflicted by that trait.

Across the five negative traits (Figure 2), including impulsivity, psychopathic tendencies, Machiavellianism/manipulativeness, narcissism, and entitlement, we found consistent evidence that people who score lower on a negative trait are more likely to believe that children today are deficient in those specific traits. These results remained the same when controlling for participants age (see Supplementary Appendix for link to results).

Thus, it is not the case that people who are high in a trait necessarily see the youth as possessing less of that trait, nor is it the case that people think the youth of today are simply lacking in all traits. People tend to believe children of the day have less of good traits on those dimensions on which they excel and more of negative traits that they avoid. Finally, as is clear in Figure 2, even the most narcissistic, the most entitled, or the most manipulative people, while holding less prejudice against the youth of the present on those traits, still don’t think kids these days are getting better. At most, individuals at each of the highs end of these trait distributions tend to think that today’s youth are similar on their respective trait to kids when they were young.

## General Discussion

Across two studies, we expanded our understanding and bounds of the persistent belief that children today are in decline. This has been referred to as the “Kids These Days” Effect (KTD) and has been shown to arise (at least in part) from a biased memory (Protzko and Schooler, 2019). People high in a trait impose their standing on the trait backward in time and apply it to children of the past. Present children naturally appear in decline compared to this artificially inflated past. Previous work demonstrated this memory mechanism through manipulating people’s beliefs in their current standing on a trait—making people feel like they were lower on a trait attenuated the KTD effect (Protzko and Schooler, 2019).

In prior research, the KTD effect was demonstrated with exclusively positive traits [e.g., intelligence, respect for authority, enjoyment of reading; Protzko and Schooler (2019)]. Here we expand this finding to negative traits, ones that are normatively considered undesirable to have, and explore another possible mechanism. First, we show people who are especially low on a negative trait are those most likely to believe that children are in decline (i.e., increasingly exhibiting) that particular trait. This relationship is consistent with the biased memory mechanism. Our second expansion was to explore an additional reason for the KTD effect. Specifically, we sought to map the belief that children are in decline onto people’s beliefs regarding how different traits grow and change over the lifespan. Our third expansion was to map how this prejudice is different for older vs. younger participants.

### Belief Children Are in Decline Over Age

In every trait we measured, the older someone was the more they believed children were deficient in that specific trait. This was equally true for thinking children had less of positive traits as well as more of negative traits. Older participants were overall more critical than younger ones.

A number of possibilities could explain older people’s particular penchant for denigrating today’s youth. If children have been continuously deteriorating over generations, then relative to younger adults, older adults would be using an objectively better reference class to compare children of the present. This seems unlikely as the same complaints have been lodged against children for millennia (e.g., Freeman, 1907). Additionally, previous work has shown the KTD effect to be at least partially independent of individuals actual experience with children in their youth, as experimentally manipulating people’s self-assessments of themselves impacts the magnitude of the KTD effect (Protzko and Schooler, 2019). Furthermore, investigations into the objective truth about some traits over generations have shown present children are actually higher on traits such as intelligence (Flynn, 1984) and the ability to delay gratification (Protzko, 2020) compared to children of the past. Therefore, the enhanced denigration of youth for older individuals is unlikely to be rooted in their accurately recalling an objectively better prior generation.

Another possible explanation for this age effect is that as people age they remember their own childhoods more favorably (Fernandes et al., 2008; cf. Field, 1997). This could lead older participants to artificially elevate their past, thinking everything was better. Accordingly, older adults’ belief that children today are particularly deficient may not reflect a reduced assessment of today’s youth so much as an inflated assessment of children from their generation. Future research should be directed at determining the exact nature of this finding.

Another factor could be that older adults may have less recent experience with contemporary youth, so they may rely more on their memory of kids from the past, and less on their experience with kids of the present.

Finally, changes in our traits as we age can also drive this age effect. As people age they become less narcissistic (Chopik and Grimm, 2019), for example. The less narcissistic someone is, the more they think children are becoming more narcissistic (study 2). Thus, as people age they may believe children are becoming more narcissistic precisely because *they* are becoming less so. Future work in the patterns of prejudice over aging could elucidate these potential mechanisms.

### Connecting Implicit Theories of Change to Denigrating Children

Across most of the traits under question, people who believed the trait does not change over the lifespan, that it is fixed, held the most moderate views toward children’s supposed decline. Across all traits but one (entitlement), those who believed the trait under question changes in any way were statistically more likely to believe children are in decline. In the case of intelligence, ability to delay gratification, ability to save money, and self-sufficiency, holding this fixed mindset was related to not only having the most moderate views toward children’s decline but also believing they were actually not in decline at all. For traits where everyone, regardless of implicit theory of change, believed children are in decline (enjoying reading, respect for authority, ability to focus, morality), holding a fixed mindset was related to having the weakest views about the decline of the youth.

The belief that stable traits show the least intergenerational decline may arise because people assume that if a trait does not change within a lifetime, then it is unlikely to change across lifetimes. Accordingly, children of the “present” are likely no different from children of the past on such an unchanging trait. This suggests that the KTD effect could be reduced by increasing people’s belief in the fixedness of traits. Unfortunately, we were not able to test this conjecture, as although we tried to manipulate this belief in Study 2, we were unsuccessful in doing so.

### Negative Traits and the Decline of the Youth

Our previously established mechanism for the belief that children are in decline was that it primarily operated through a memory bias (Protzko and Schooler, 2019). This occurs because people who were high in a positive trait (like intelligence or being well-read) impose that backward in time onto children of their youth, artificially exaggerating their possession of that trait. In reality, our memories for what all children were like decades ago is not accurate. This memory bias makes the interesting prediction that those who are high in a negative trait (e.g., narcissism) impose that backward in time to all children (e.g., all children of the past were narcissistic) and then compared to an artificially exaggerated memory, children today appear *less* narcissistic. This is exactly what we found with the negative traits. In study 2, every negative trait showed this same pattern, namely, those high in a negative trait held weaker beliefs about the decline of the youth than those who were low on the negative trait.

It is notable that, on average, people did not believe the youth of the day were getting better on any trait than previous generations. This is especially noteworthy because some traits we investigated, like the ability to delay gratification, have been increasing over generations (Protzko, 2020). The memory model that helps explain these prejudices and why they are stronger among some people assumes that people have some sense of their quality on a given dimension. Possessing some knowledge of their particular traits enables individuals to project those qualities back on to all children of their generation. A corollary of this account is that any biases in self-appraisal should similarly be projected back. Indeed people’s positive bias regarding their own self-assessments [e.g., Zell et al. (2020)] may help to explain why people who excel in positive traits perceive kids these days as in decline on those traits, whereas people who excel on negative traits at best think kids today are comparable to kids of the past. Accordingly, when people excel on a positive trait their inflated assessment is passed on to their appraisal of children in the past, thereby causing them to think today’s children are lacking. When people are lacking in a positive trait or excel on a negative trait, their upward assessment of themselves is similarly passed on to the children of their generation, causing them to be perceived as at least average on that trait and thus no different from average children today.

### Limitations

One limitation of these studies is we did not assess whether participants were parents themselves. It could be the case that parents have a different view of the “kids these days” phenomenon themselves. Having more experience with today’s youth, parents might have a more accurate assessment and thus exhibit a reduced KTD effect. Alternatively, parents might be more inclined to think back to when they were kids, thereby inflating the memorial mechanism that has been found to drive the KTD effect. Future work should include these important moderators for investigation.

## Conclusion

For millennia, people have believed the youth of the day are in decline compared to previous generations. While this work cannot speak to the veracity of claims that children are more or less narcissistic or entitled or manipulative, we can now better understand why it may appear so strongly to some people and not to others. First, older people are more likely to see this supposed decline. Second, belief in whether a trait changes over the lifespan is associated with such prejudices. Holding a fixed mindset about a trait is associated with showing the least amount of intergenerational prejudice toward the youth. Finally, this supposed decline occurs similarly for both positive and negative, with present youth being attributed both less of positive traits and more of negative traits. The higher one is on a positive trait, the less of that trait one sees the youth having. Conversely, the lower one is on a negative trait, the more one sees the youth of today as afflicted by that trait. Prejudice against the youth of the day entails them both not having the good traits we have, and possessing the bad traits we do not. It seems the age-old tendency to denigrate the youth of the present will continue until we recognize that their apparent decline is not a failing of them, but of our memories of what kids were like in the past.

## Data Availability Statement

The datasets presented in this study can be found in online repositories. The names of the repository/repositories and accession number(s) can be found in the article/Supplementary Material.

## Ethics Statement

The studies involving human participants were reviewed and approved by ORAHS, University of California, Santa Barbara. The patients/participants provided their written informed consent to participate in this study.

## Author Contributions

JP collected the data, ran the analyses, and wrote the manuscript. Both authors developed the concept, designed the study, edited the final version, and approved the submitted version.

## Conflict of Interest

The authors declare that the research was conducted in the absence of any commercial or financial relationships that could be construed as a potential conflict of interest.

## Publisher’s Note

All claims expressed in this article are solely those of the authors and do not necessarily represent those of their affiliated organizations, or those of the publisher, the editors and the reviewers. Any product that may be evaluated in this article, or claim that may be made by its manufacturer, is not guaranteed or endorsed by the publisher.
