# Supplementary material for: Who Denigrates Today’s Youth?: The Role of Age, Implicit Theories, and Sharing the Same Negative Trait
Source: Front Psychol. 2022 May 30;13:723515. doi: 10.3389/fpsyg.2022.723515 (PMC9192333; doi:10.3389/fpsyg.2022.723515)
Supplement: Supplementary file 1 [file Table_1.DOCX]

**Manipulating Implicit Theories of Change**

Afterwards, we attempted to manipulate implicit theories of change. We chose manipulating implicit theories of change for respecting authority, attempting to move people from their most common belief (it increases throughout the lifespan) to one related to holding significantly less disfavorable views towards children (it is flat and does not change throughout the lifespan).

Participants read the same instructions for selecting the implicit theory of change as in study 1. Instead of the full nine graphs, however, we only showed increasing, decreasing, or flat options. They were also given the same comprehension check as in study 1, this time with only the three pictures and asked to select one showing a decrease in a trait across the lifespan. Next, participants were randomly assigned to one of three conditions, flat, increasing, or a control. Those in the flat or increasing condition read:

“Next we are going to show you an article about respect for authority. Please take a couple of minutes are read the article thoroughly. We will ask you some questions about the article afterwards to make sure you have read it.”

Participants then read an article about the genetics of respect for authority and how it is stable (flat condition) or increases (increasing condition) throughout the lifespan. This article was taken from a real press release and adjusted by us to fit the needs here (see <https://osf.io/j93wz/> for the files).

Participants were then administered an attention check:

“According to the article you just read, how does the trajectory of respect for authority look like throughout the lifespan?”

with the increasing, decreasing, or flat graph. Afterwards, participants were asked the kids these days: respect question:

“Compared to when you were a child: Do you think children today are more respectful of elders, less respectful, or equally respectful as children were when you were a child?”

Participants in the control condition first filled out the kids these days: respect scale and then the article instructions and were randomly given one of the article to read. Afterwards, all participants filled out a seriousness check (Aust et al., 2013) and a captcha.

**Results**

**Intervention Effect on Implicit Theories**

First, 64% of the sample passed the initial comprehension check (65% of all those who also passed the seriousness check). Of those, 85% complied with the intervention (83% when including everyone); meaning they indicated the correct pattern of change the article indicated respect for authority exhibits. Our primary analysis was an intention to treat design, where participants are analyzed in their condition regardless of whether they complied with the intervention.

For the overall intervention effects, there was no difference in how much people believed children today no longer respect authority based on what article they read (*F*(2, 917) = .01, *p* = .988). Our primary concern was the difference between those induced to believe respect for authority increases throughout the lifespan versus those who were induced to believe respect for authority is flat across the lifespan. This analysis revealed no significant effect of the intervention on beliefs (*b* = .035, *p* = .752), regardless of if they complied (*b*_iv_ = 2.084, *p* = .571). Although we were unable to successfully manipulate the belief that children today are less respectful through manipulating the relevant implicit theory of change, we did discover a breadth of new traits, positive and negative, where ‘present’ children are believed to be worse than previous generations.
