# Supplementary material for: Who Denigrates Today’s Youth?: The Role of Age, Implicit Theories, and Sharing the Same Negative Trait
Source: Front Psychol. 2022 May 30;13:723515. doi: 10.3389/fpsyg.2022.723515 (PMC9192333; doi:10.3389/fpsyg.2022.723515)
Supplement: Supplementary file 2 [file Presentation_1.pdf]

Implicit Theories of Change over the Lifespan

People hold very different implicit theories of change for different traits. No single implicit theory was the most common over all ages. Furthermore, the correlation between people on beliefs was very small, ranging from $r_s = .066$ to $r_s = .204$. This suggests that across traits people do not hold the same implicit theory of change. Therefore, we need to understand the beliefs of change on a per-trait basis as well as looking over ages.

To do this, we model what would constitute the *a* path in a typical mediation design. This looks at the relationship of holding different implicit theories of change with age. The possibilities for implicit theories of change were again that over the lifespan a trait: increases, decreases, is flat, is U-shaped, declines in the elderly, is parabolic, plateaus in adulthood, declines to a floor in adulthood, and experiences a spike in the elderly (replicating Ross, 1989).

Intelligence

The modal belief across participants was intelligence increases throughout the lifespan (23% of participants holding this belief). This belief in increasing intelligence, however, becomes less likely as we age ($b = -.002$, $p = .004$, 95%CI = -.004 to -.001). In the elderly, belief in constant intellectual progress is replaced by the growing belief that it is flat across the lifespan ($b = .002$, $p < .001$, 95%CI = .003 to .001). Other results show that the implicit theory that intelligence is parabolic likewise decreases across the lifespan ($b = -.002$, $p = .024$, 95%CI = -.003 to -.002). Finally, over age, there is a small growing belief that there is a sudden decrease in the elderly ($b = .001$, $p = .043$, 95%CI = .002 to < .001l see **Figure S1**).

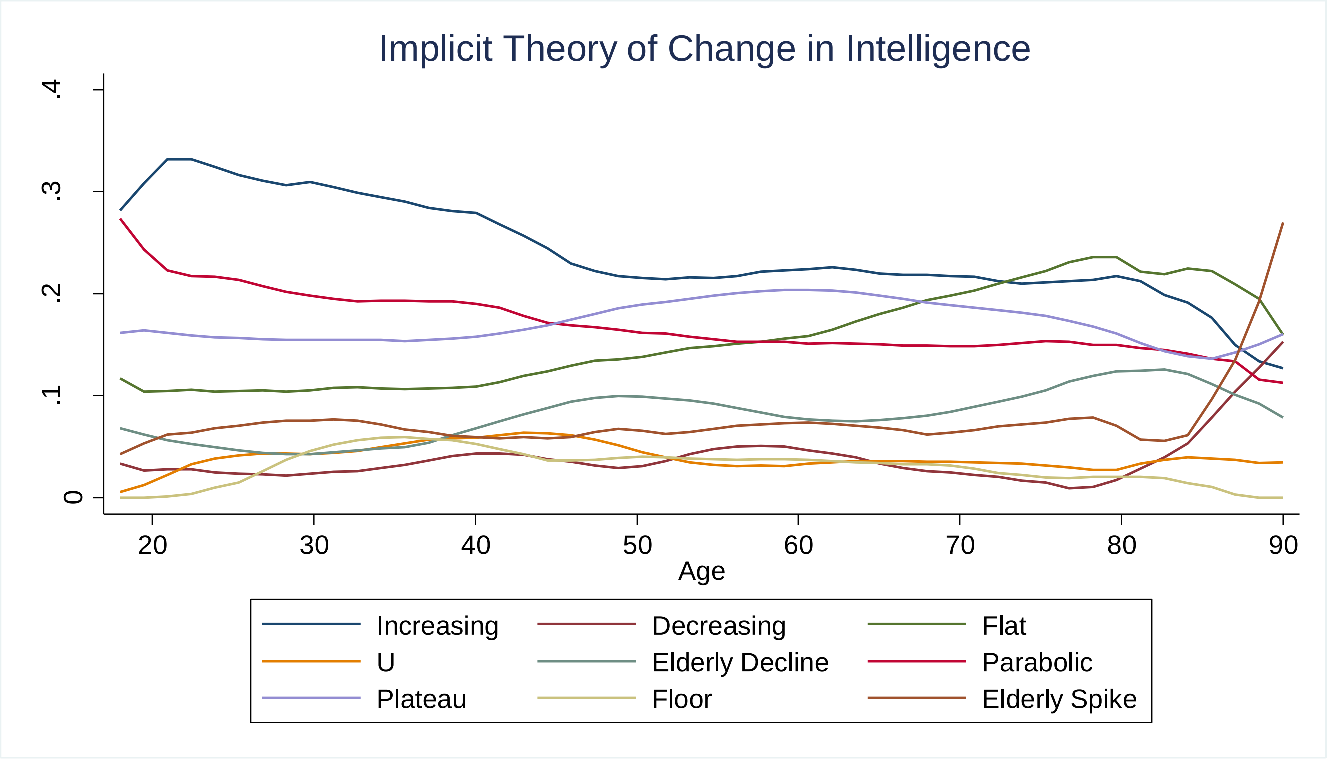

Figure S1: Marginal predicted probabilities showing the likelihood of choosing each intuitive theory of change over the lifespan for intelligence. Lines are kernel-weighted local polynomial smoothed lines to display non-linear trends.

Enjoying Reading

Across all ages, the most common implicit theory of change for enjoying reading across the lifespan was flat stability (19%, which did not change over age (*p* = .897)). As people age, they were less likely to believe enjoyment of reading declines (*b* = -.001, *p* = .042, 95%CI = -.001 to < .001) or that it followed the 2nd most popular belief, following a U-shaped pattern (*b* = -.001, *p* = .035, 95%CI = -.032 to -.001) and less likely to endorse the linear decline (*b* = -.01, *p* = .033, 95%CI = -.003 to <-.001). These declines were replaced with an increasing belief with

ageing that there is a sudden increase in enjoying reading in the elderly (b = .001, p = .02, 95%CI = .002 to <.001; **Figure S2**).

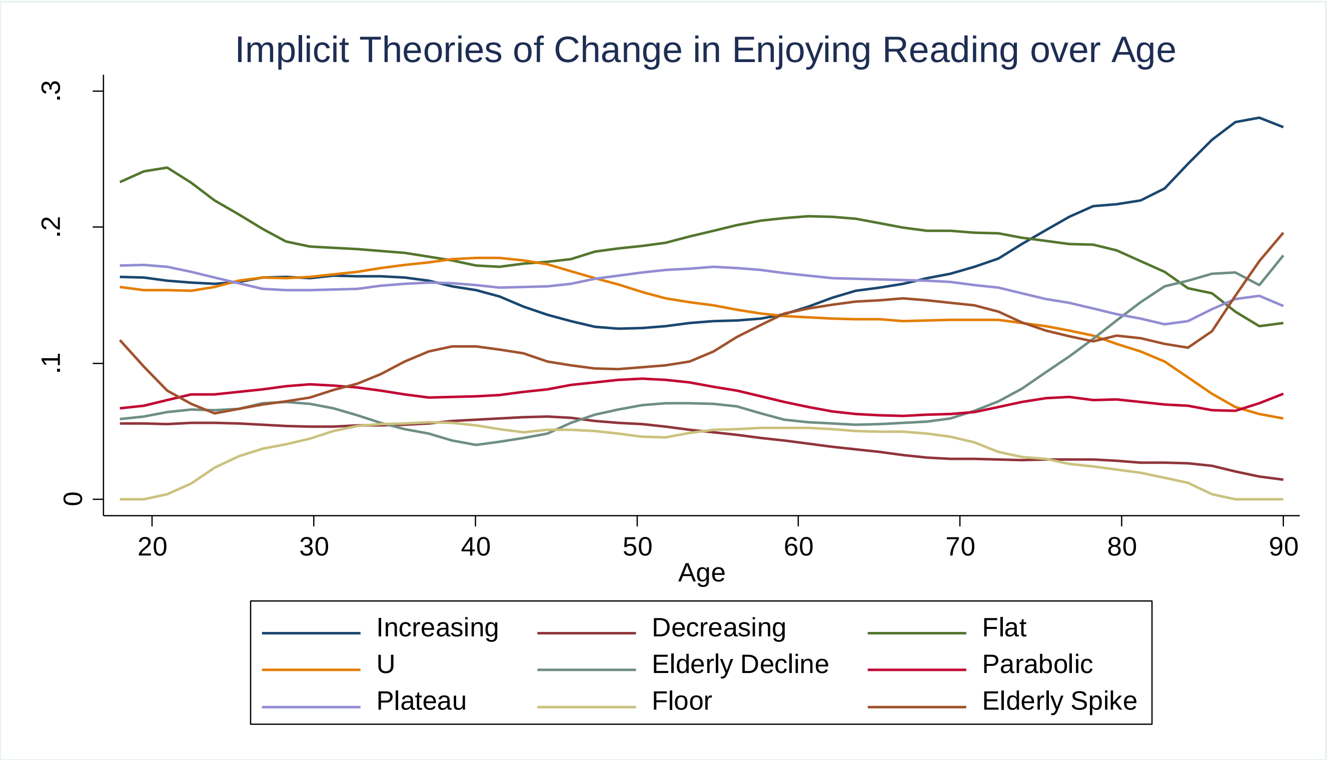

Figure S2: Marginal predicted probabilities showing the likelihood of choosing each intuitive theory of change over the lifespan for enjoying reading. Lines are kernel-weighted local polynomial smoothed lines to display non-linear trends.

Respect for Authority

The most common belief was that respect for authority increases linearly as we age (22%, which did not significantly change over age (p = .147)). The only significant change in implicit theories was with age there was an increase in believing respect for authority is stable through life until elderly where there is an upward spike (b = .001, p = .028, 95%CI = .003 to < .001; Figure S3).

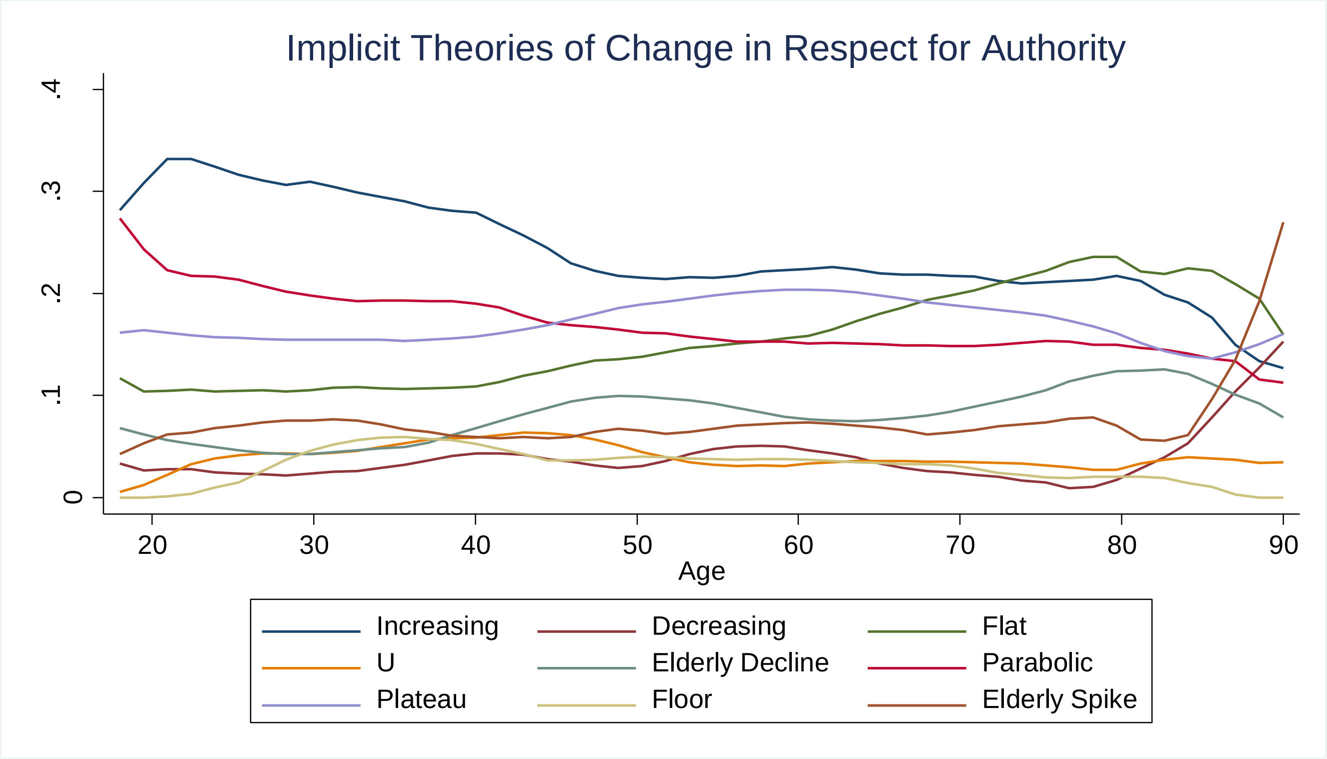

Figure S3: Marginal predicted probabilities showing the likelihood of choosing each intuitive theory of change over the lifespan for respect for authority. Lines are kernel-weighted local polynomial smoothed lines to display non-linear trends.

Ability to Focus

The most common implicit theory was the ability to focus on just one thing shows a parabolic trajectory over the life course increasing through childhood into adulthood but then worsening as we turn elderly (24%; with no change over age: p = .227). While there were a number of implicit theories showing non-significant decreases over age, as people aged they believed a sudden decrease in the elderly's ability to focus occurred (b = .001, p = .035, 95%CI = .003 to < .001; Figure S4).

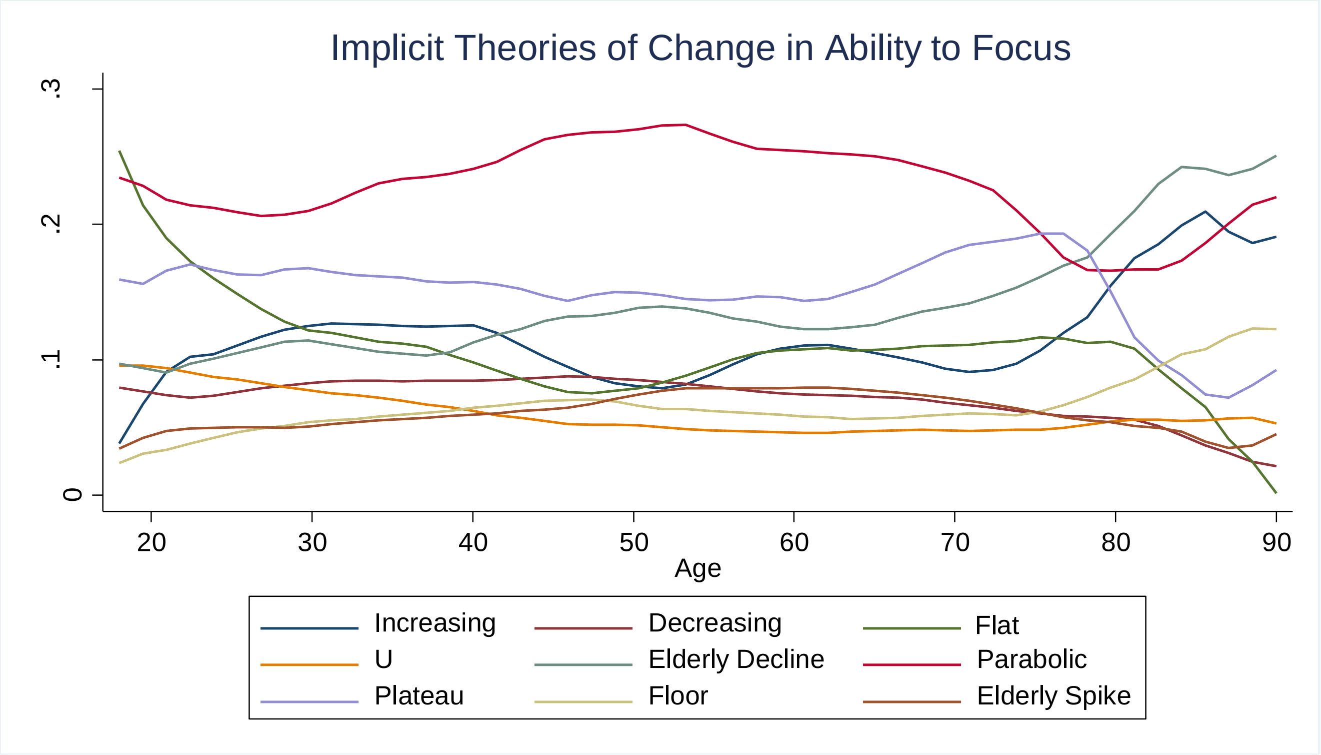

Figure S4: Marginal predicted probabilities showing the likelihood of choosing each intuitive theory of change over the lifespan for the ability to focus on one thing. Lines are kernel-weighted local polynomial smoothed lines to display non-linear trends.

Delaying Gratification

The most common implicit theories about the ability to delay gratification were either that it increased throughout the lifespan (19%) or that increased through childhood and plateaued for the rest of life (19%); neither of these beliefs showed any change over age (*p*s = .507 & .426, respectively). What did change was as people get older they are less likely to believe the ability to delay gratification is unchanging throughout life (*b* = -.002, *p* = .002, 95%CI = -.003 to -.001), being replaced by an increasing belief that there is a sudden spike in the elderly (*b* = .002, *p* = .001, 95%CI = .003 to .001; Figure S5).

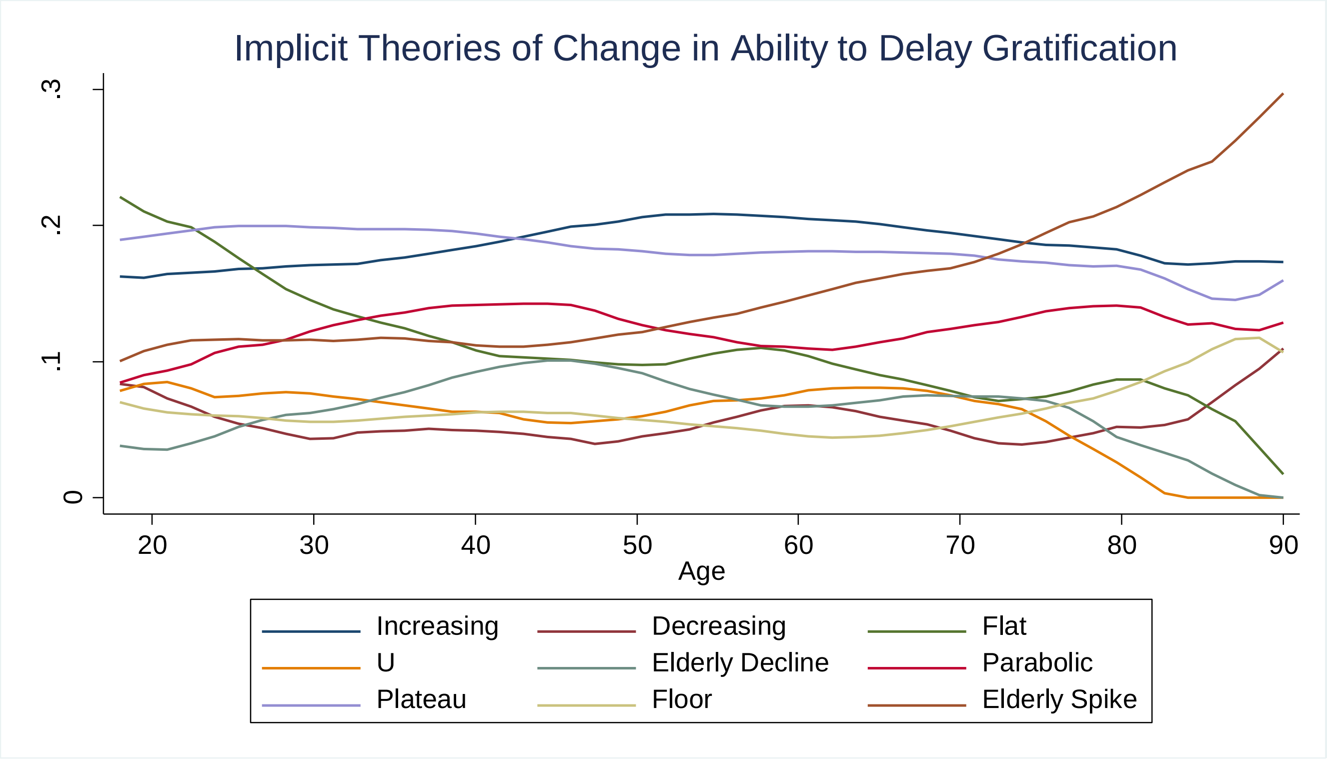

Figure S5: Marginal predicted probabilities showing the likelihood of choosing each intuitive theory of change over the lifespan for the ability to delay gratification. Lines are kernel-weighted local polynomial smoothed lines to display non-linear trends.

Morality

The most common implicit theories about morality was that it was constant and unchanging throughout the lifespan (20%; showing no change over age (*p* = .206)). While there were a number of implicit theories showing a non-significant increase, both the belief that morality increases throughout the lifespan (*b* = -.002, *p* = .002, 95%CI = -.003 to -.002) and that it decreases throughout the lifespan (*b* = -.001, *p* = .012, 95%CI = -.002 to <-.001) both showed significant reductions as people aged (Figure S6).

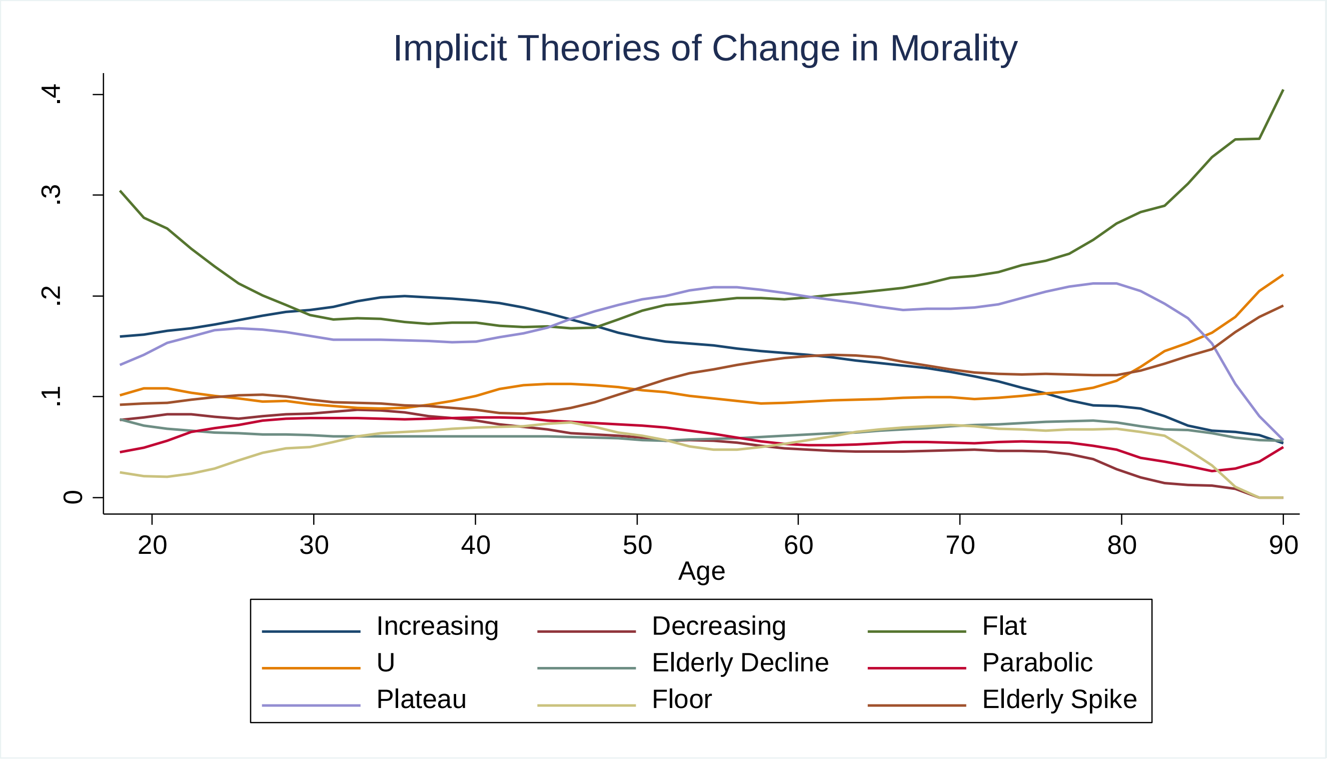

Figure S6: Marginal predicted probabilities showing the likelihood of choosing each intuitive theory of change over the lifespan for being morally good. Lines are kernel-weighted local polynomial smoothed lines to display non-linear trends.

Ability to Save Money

The two most common implicit theories were that the ability to save money is constant until it spikes in old age (19%) and that the ability to save money increases throughout the lifespan (18%). While the increase-implicit theory did not show a significant decline over age (*p* = .113), the belief in the sudden elderly spike increased throughout ageing (*b* = .002, *p* = .005, 95%CI = .003 to .001). Furthermore, older people were more likely to believe the elderly show a sudden drop in the ability to save money as well (*b* = .001, *p* = 003, 95%CI = .002 to < .001; Figure S7).

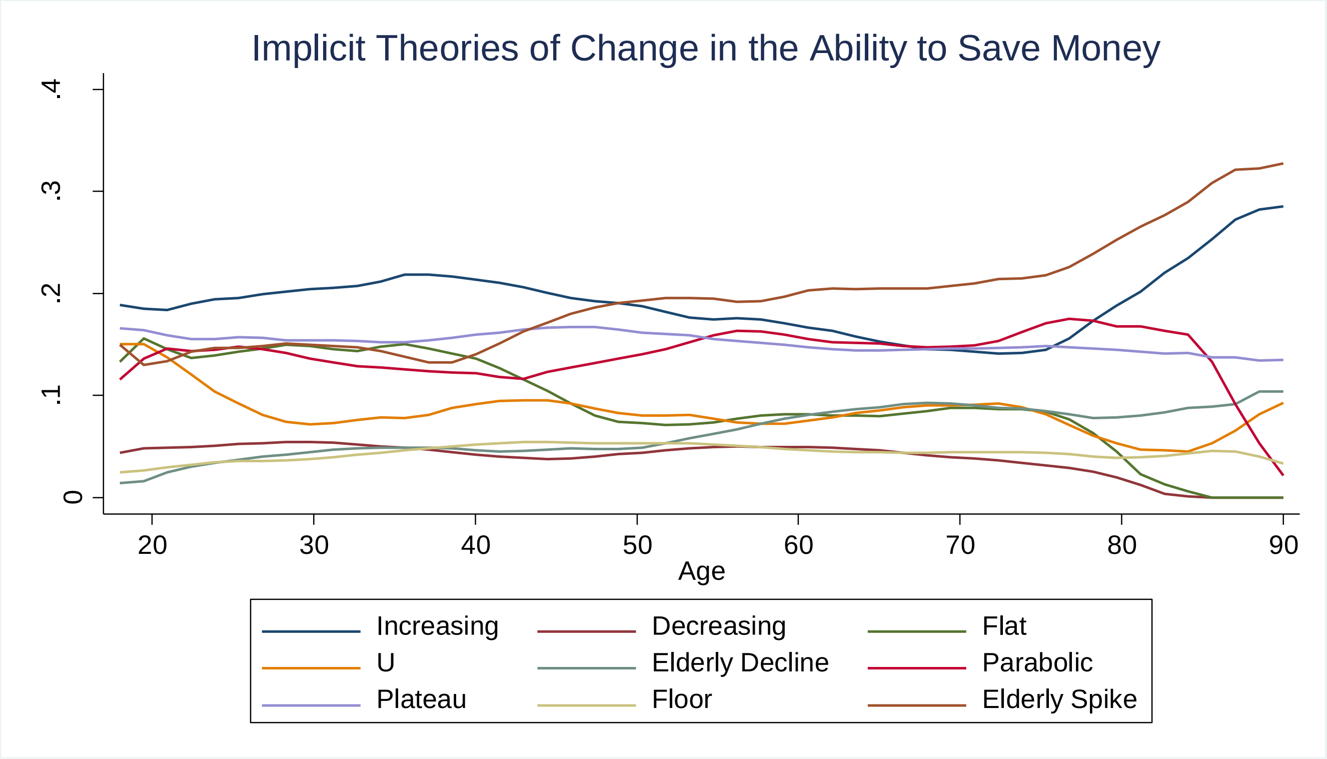

Figure S7: Marginal predicted probabilities showing the likelihood of choosing each intuitive theory of change over the lifespan for the ability to save money. Lines are kernel-weighted local polynomial smoothed lines to display non-linear trends.

Work Ethic

The most common belief was that work ethic increases throughout childhood where it then plateaus for the rest of life (24%); a belief that shows no change over age (p = .488). The least likely option at all ages, that of a decrease from childhood through the elderly, became even less endorsed as people aged (b = -.001, p = .043, 95%CI = -.001 to < .001). As people became older, they became more likely to believe that work ethic showed either a flat unchanging pattern of development (b = .002, p = .005, 95%CI = .003 to .001), or there was a spike in work ethic in old age (b = .001, p = .02, 95%CI = .002 to < .001; Figure S8).

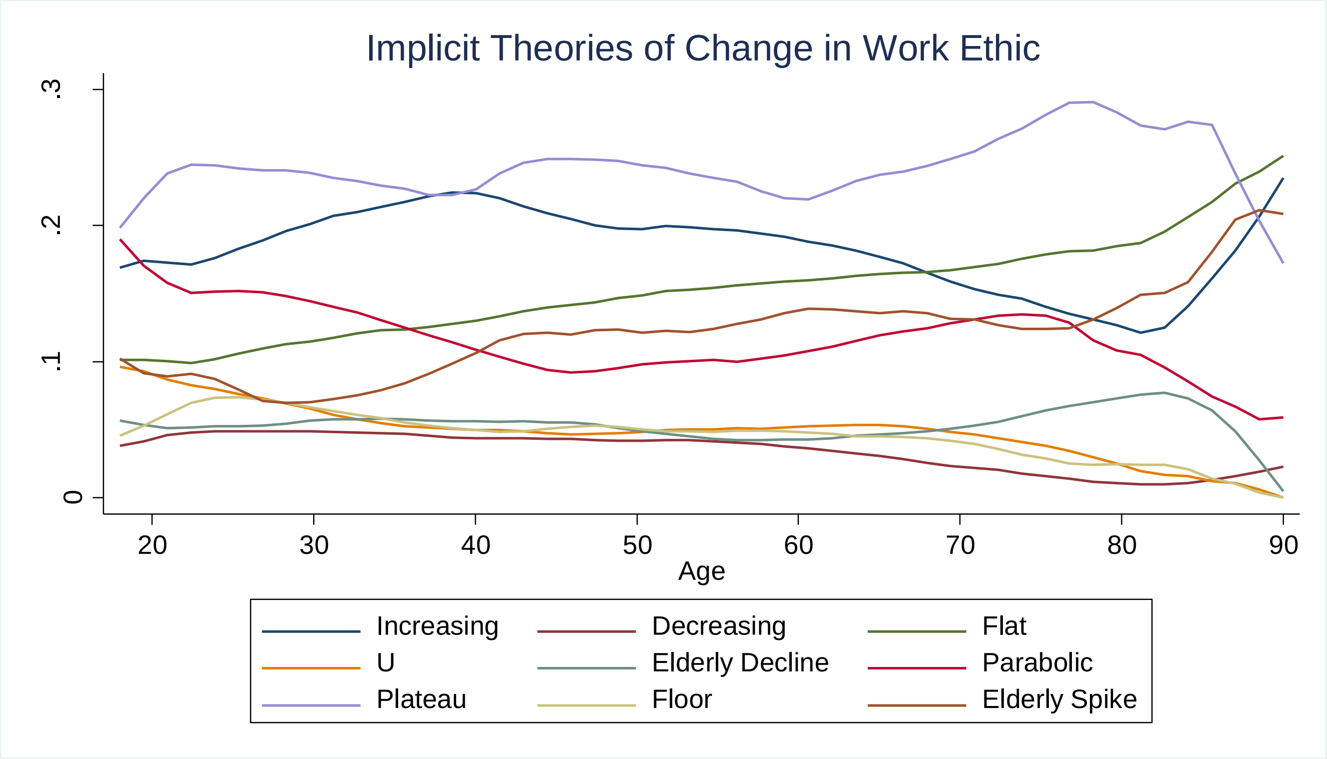

Figure S8: Marginal predicted probabilities showing the likelihood of choosing each intuitive theory of change over the lifespan for work ethic. Lines are kernel-weighted local polynomial smoothed lines to display non-linear trends.

Self-sufficiency

The most common implicit theory was that self-sufficiency is parabolic (35%), starting off low, increasing through life to adulthood, the dropping again in the elderly; this was seen across all ages with no change (*p* = .379). The only change seen over age is that as people get older they become more likely to believe there is a sharp drop in self-sufficiency in old age (*b* = .001, *p* = .031, 95%CI = .002 to < .001; Figure S9).

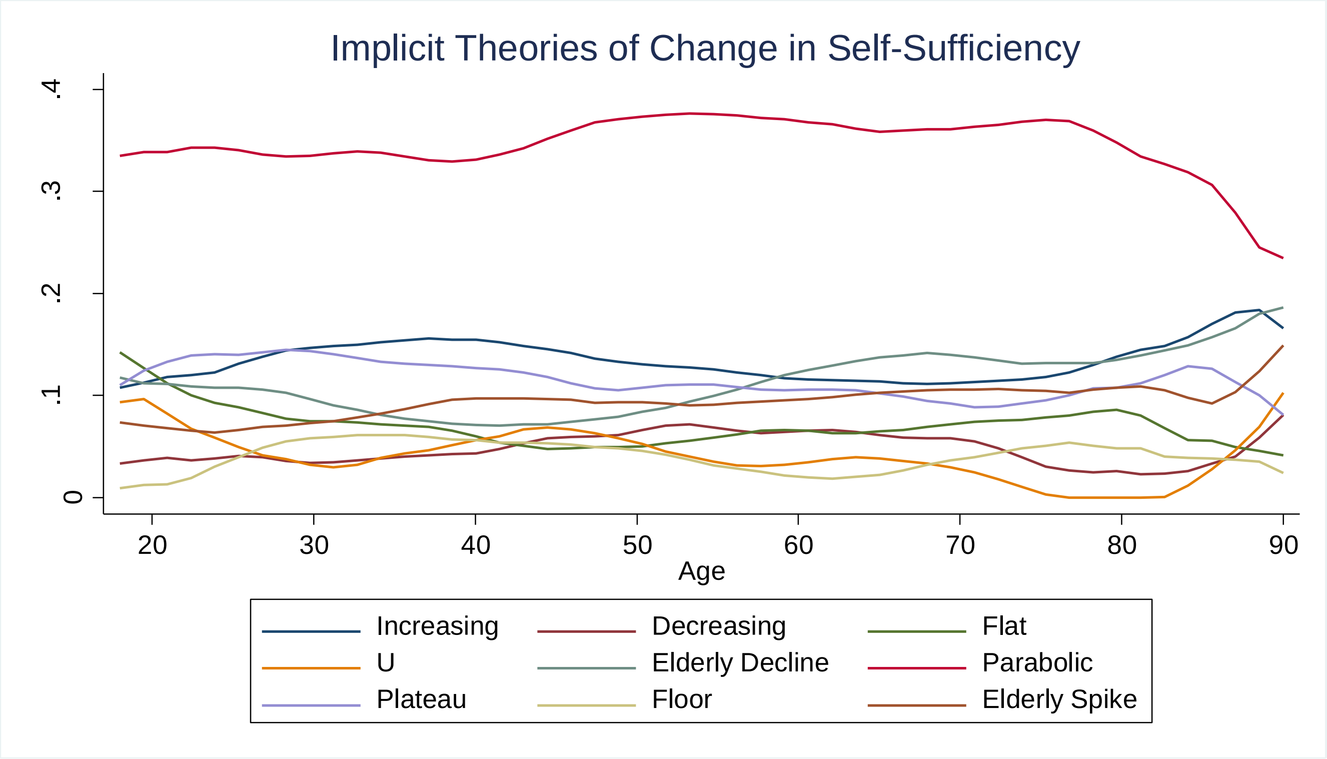

Figure S9: Marginal predicted probabilities showing the likelihood of choosing each intuitive theory of change over the lifespan for being self-sufficient. Lines are kernel-weighted local polynomial smoothed lines to display non-linear trends.

Entitled

Implicit theories of change exhibited nearly a complete reversal over age. No one implicit theory was endorsed more than the others at all ages. And while most beliefs declined non-significantly, two patterns are noteworthy. The belief that entitlement shows stability until the late life where it sharply declines was the 2nd least endorsed by younger participants but most endorsed by older participants (b = .002, p = .006, 95%CI = .003 to <.001). Also, the belief that entitlement is stable but in the elderly has a sharp spike upwards was among the least endorsed by younger participants but by old age was the 2nd most endorsed belief (b = .002, p = .005, 95%CI = .003 to <.001; Figure S10).

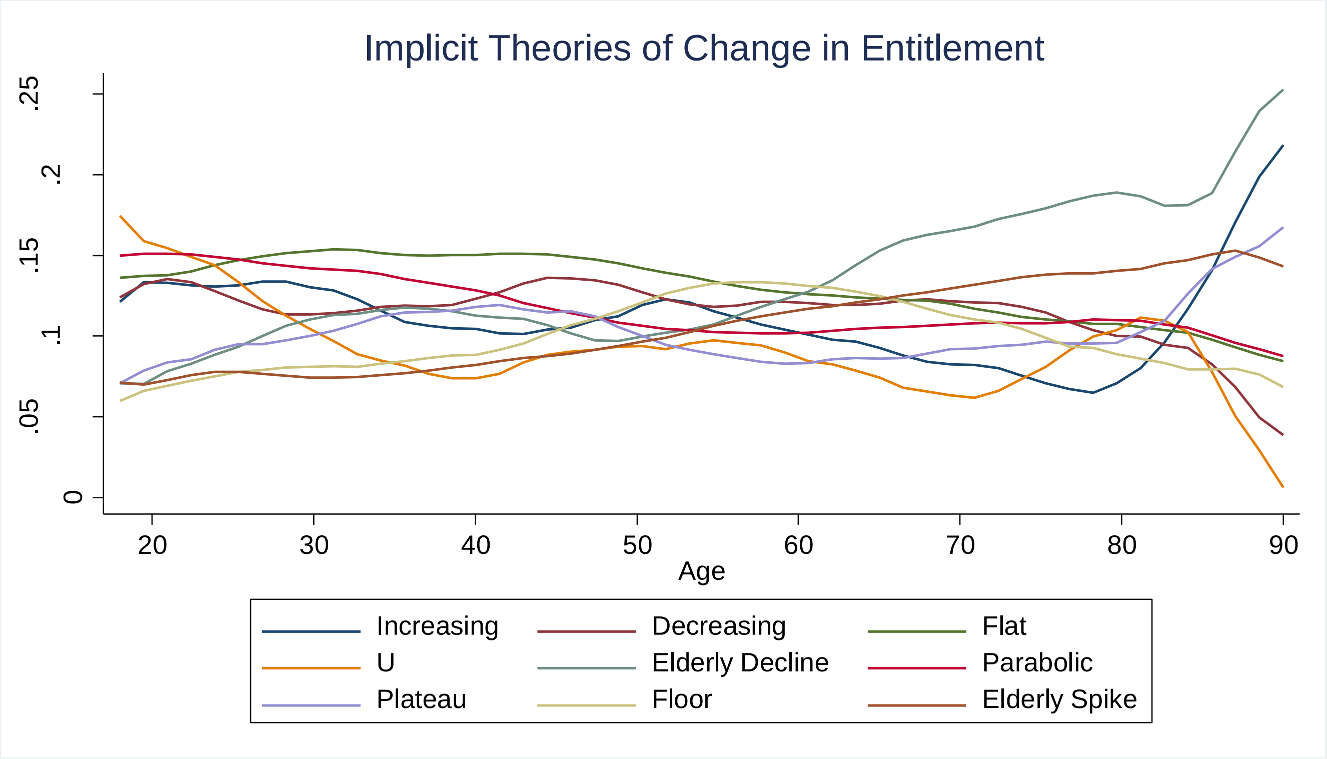

Figure S1: Marginal predicted probabilities showing the likelihood of choosing each intuitive theory of change over the lifespan for being entitled. Lines are kernel-weighted local polynomial smoothed lines to display non-linear trends.

Discussion

Implicit Theories of Change over Age

What people think about how a trait changes over the lifespan may be idiosyncratic to the trait under question. No single implicit theory of change was consistently most endorsed across all traits under investigation, and the correlations within people over traits was small. Within each trait, there was a large amount of heterogeneity in how people thought of the development of a trait. Most importantly, however, these beliefs were not constant across ages.

The most common implicit theories of change involved some form of growth and development from childhood into adulthood—before either continuing to increase (as with

respect for authority, delay of gratification, saving money), tapering off (as with intelligence, morality, work ethic) or declining (as with ability to focus, self-sufficiency).

As people age, their personal experiences with the traits under question may influence their implicit theories. The most common belief about intelligence, for example, was that it increases throughout the lifespan, growing from childhood to adulthood to the elderly. As people aged, however, they became less likely to endorse this pattern, and more likely to believe a pattern that involved decline in the elderly. This likely occurs as people experience first-hand the negative effects of the ageing process on one’s cognitive abilities (e.g. Schaie, 2005). Future work can build off these findings to explore the causes of changes in implicit theories.

Supplemental References

Schaie, K. W. (2005). *Developmental influences on adult intelligence: The Seattle longitudinal study*. Oxford University Press.
